# In search of a primary outcome for community-based newborn infection trials in Eastern Uganda: a nested cohort study within the BabyGel pilot trial

**DOI:** 10.1186/s40814-019-0428-3

**Published:** 2019-03-13

**Authors:** N. A. Mobbs, J. Ditai, J. Abeso, E. B. Faragher, E. D. Carrol, M. Gladstone, A. Medina-Lara, P. Olupot-Olupot, A. D. Weeks

**Affiliations:** 1 Sanyu Research Unit, Department of Women’s and Children’s Health, University of Liverpool and Liverpool Women’s NHS Foundation Trust, members of Liverpool Health Partners, Crown Street, Liverpool, L8 7SS UK; 2 University of Liverpool, Cedar House, Ashton Street, Liverpool, L3 5PS UK; 3 Sanyu Africa Research Institute (SAfRI), Mbale Regional Referral Hospital, Pallisa-Kumi Road Junction, P.o Box 2190, Mbale, Uganda; 4 Department of Paediatrics, Mbale Regional Referral Hospital, Mbale, Uganda; 5 Tropical Clinical Trials Unit, Liverpool School of Tropical Medicine, Pembroke Place, Liverpool, L3 5QA UK; 6 Department of Clinical Infection, Microbiology and Immunology, Institute of Infection and Global Health, University of Liverpool, 8 West Derby Street, Liverpool, L69 7BE UK; 7 Department of Women’s and Children’s Health, Institute of Translational Medicine, University of Liverpool and Alder Hey NHS Foundation Trust, members of Liverpool Health Partners, Eaton Road, Liverpool, L12 2AP UK; 8 Health Economics Group, University of Exeter, Exeter, UK; 9 Faculty of Health Sciences, Busitema University, P.o Box 1460, Mbale, Uganda

**Keywords:** Paediatrics, Neonate, Infection, Uganda, Primary outcome, Diagnosis, Hand hygiene, Sepsis, Antibiotics

## Abstract

**Background:**

Due to their immature immune system, neonates are at high risk of infection. This vulnerability when combined with limited resources and health education in developing countries can lead to sepsis, resulting in high global neonatal mortality rates. Many of these deaths are preventable. The BabyGel pilot trial tested the feasibility of conducting the main randomised trial, with the provision of alcohol handgel to postpartum mothers for prevention of neonatal infective morbidity in the rural community. This secondary analysis sought to evaluate the methods of detecting infections in babies up to 3 months of age.

**Methods:**

The pilot two-arm cluster randomised controlled trial took place in 10 villages around Mbale, Eastern Uganda. Women were eligible and recruited antenatally if their gestation was ≥ 34 weeks. All infants of mothers participating in the BabyGel pilot trial were followed up for the first 3 months of life. Evidence for infant infection was collected using five different methods: clinician diagnosed infection, microbiologically confirmed infection, maternally reported infection, a positive infection screen using the World Health Organization (WHO) Integrated Management of Childhood Illness (IMCI) screening criteria, and reported antibiotic use identified during home and clinic visits. These methods were assessed quantitatively regarding the detection rates of suspected infections and qualitatively by exploring the challenges collecting data in the rural community setting.

**Results:**

A total of 103 eligible women participated in the BabyGel pilot trial, with 1 woman delivering twins. Of the 99 mother-infant pairs who consented to participate in the study, 55 infants were identified with infection in total. Maternal report of illness provided the highest estimate, with mothers reporting suspected illness for 45 infants (81.8% of the total suspected infections identified). The WHO IMCI screening criteria identified 30 infants with suspected infection (54.5%), and evidence for antibiotic use was established in 22 infants (40%). Finally, clinician-diagnosed infection identified 19 cases (34.5%), which were also microbiologically confirmed in 5 cases (9.1%). Data collection in the rural setting was hindered by poor communication between mothers and the research team, limited staff awareness of the study in health centres resulting in reduced safeguarding of clinical notes, and widespread use of antibiotics prior to notification and clinical review. Furthermore, identification of suspected infection may not have been limited to severe infections, with ambiguity and no official clinical diagnosis being given to those identified solely by maternal report of infection.

**Conclusions:**

A high rate of suspected infection was identified spanning the five sources of data collection, but no ideal method was found for detection of community neonatal infection. Although maternal self-reports of infant infection provided the highest detection rate, data collection via each source was limited and may have identified minor rather than major infections. Future studies could utilise the IMCI screening tool to detect severe community infection leading to referral for clinical confirmation. This should be combined with weekly contact with mothers to detect maternally suspected illness. Obtaining more details of the symptoms and timescale will improve the accuracy when detecting the total burden of suspected disease, and advising participants to retain medication packaging and prescriptions will improve identification of antibiotic use.

**Trial registration:**

Babygel pilot trial - trial registration: ISCRCTN 67852437. Registered 02/03/2015.

## Introduction

Neonatal sepsis remains a global problem, causing 15% of all neonatal deaths [1] and claiming over 1.5 million infant lives annually [2]. These statistics may even be higher as neonatal mortality in developing countries is potentially under-reported by > 20% [3]. During the first year of life, an underdeveloped immune system renders infants particularly vulnerable to infection [1, 4, 5]. This vulnerability is further exacerbated in developing countries, such as Uganda, by limited resources and awareness. Despite the global burden of neonatal deaths declining, the African region still holds the highest neonatal mortality rate (NMR), with 30.5 per 1000 live births [6]. Since the initiation of the Millennium Developmental Goals, Sub-Saharan Africa has witnessed the largest relative decrease in under 5 mortality, yet still carries the burden of half the world’s under 5 deaths and the proportion of those deaths within the first year of life increased [7]. The statistics highlight a demographic which drastically demands attention. High birth rates combined with high-risk newborn care practices and unhygienic environments including open rubbish dumps, roaming animals, and standing water may contribute to the high levels of morbidity and mortality. These settings with proportionately high and arguably preventable neonatal fatalities are those where affordable, acceptable, and sustainable interventions could yield the greatest and most immediate gains [1].

Low household wealth, lack of maternal education, and birth in rural settings are considered three critical determinants of NMR. A recent study in Eastern Uganda found the NMR to be higher in rural districts: 34 per 1000 compared with the national NMR of 20 per 1000 (2013) [8, 9]. In Uganda, 42% of rural births are unattended [10], and combined with lack of sanitary equipment, limited access to clean water, potential lack of education, and unhygienic practices, risk of infection is high [11, 12]. As many rural communities lack disposable amounts of clean water for hand washing, alcohol hand rub may offer the potential to reduce the rates of neonatal sepsis and improve the status of Africa’s NMR [13].

The BabyGel study [ISRCTN67852437] (reported elsewhere) targeted a reduction in intrapartum and postnatal infection transmission by distributing alcohol hand rub to pregnant women antenatally in rural communities of Uganda for use during the birth and for 3 months postnatally [14]. The intervention aimed to prevent early-onset sepsis due to intrapartum maternal transmission and late-onset sepsis via human transmission of environmental pathogens.

Studies documenting rural community-acquired infection in neonates in Sub-Saharan Africa are scarce, largely due to the difficulties in data collection. No major studies have been done to identify rates of community-acquired infection including sepsis in rural Uganda [15, 16], despite 88% of the population residing in rural areas [10]. The only related studies conducted in Uganda’s Mbarara Regional Referral Hospital and Mulago Hospital aimed to identify pathogenic organisms cultured from hospitalised septic neonates [2, 12]. Both studies found *Staphylococcus aureus* and *Escherichia coli* were the most common causative pathogens identified. Overall, the range of positive cultures was limited; 65% and 63% of pathogens were unidentifiable in Mbarara and Mulago hospitals respectively [2]. These limited results are reflected in similar studies in developing countries globally [17–20]. The obstacle to identifying the range of harmful pathogenic organisms is rooted in the fact that investigation strategies in the developing world are limited to blood and cerebrospinal fluid (CSF) cultures. Polymerase chain reaction (PCR) methods may yield greater results, but are generally unavailable [21, 22].

Although microbiological confirmation of infection is the gold standard for identifying neonatal sepsis, this cannot always be achieved in developing communities and diagnosis is often based solely on clinical signs [23]. Based on this, the Young Infants Clinical Signs Study Group (YICSSG) identified seven clinical signs which could be used with high sensitivity and specificity to predict requirement for neonatal referral, and WHO integrated this diagnostic screening tool in their guideline for the Integrated Management of Childhood Illnesses [24, 25]. The validated IMCI screening form and diagnostic signs have since been adapted, utilised, and tested in many developing countries globally in rural settings to identify suspected infection [26, 27]. This screening tool was utilised in the BabyGel study to assess infectious morbidity.

This paper reports a secondary analysis of the pilot trial, comparing the various methods for identifying suspected infection in the initial 3 months of life in rural Ugandan communities.

## Methods

### Study setting and participant recruitment

The BabyGel pilot trial was conducted in 10 villages around Mbale, Uganda. Consent was sought from all mothers who were estimated to be at > 34 weeks’ gestation at the time of recruitment and resided in a participating village. A 3-month recruitment period was considered adequate by the investigators to assess the ability to recruit women to the study and resulted in recruitment of 103 women (who went on to deliver 104 newborns).

### Study timeline

A timescale of 3 months was chosen to follow up the newborns/infants. This was chosen as a balance between the duration of time over which the provision of alcohol hand rub could be sustained and the period during which neonatal infection rates are highest and infant immunity is lowest. Vulnerability to infection is greatest during that early period, with one third of all child deaths occurring during the initial month of life [28].

### Data collection

The gamut of suspected infection was identified using five methods of data collection: community-based IMCI screening forms, clinician-diagnosed infection detailed in hospital notes, microbiologically confirmed infection, and maternally reported infection and antibiotic use. The data collected was utilised to calculate the burden of disease amongst the participating infants in both the control and intervention groups.

**Fig. 1:**
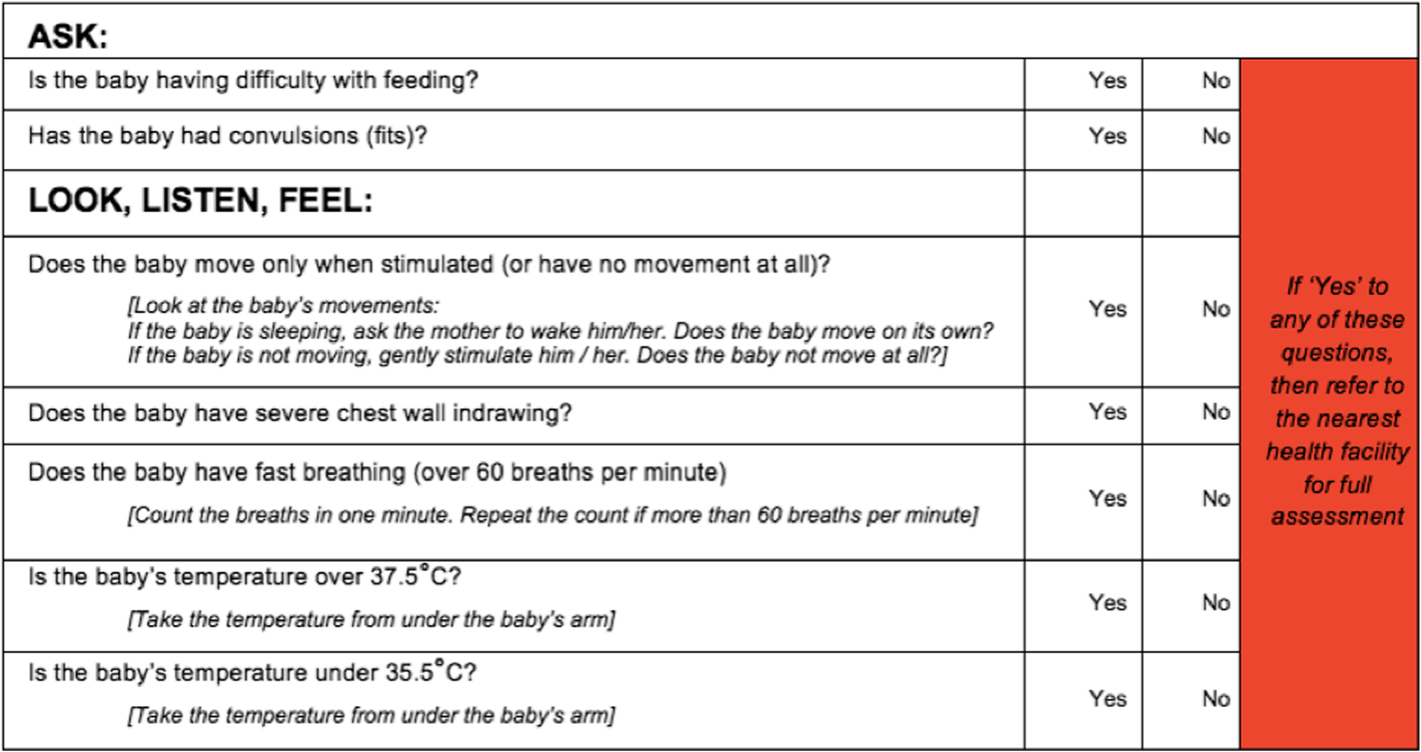
The IMCI screening criteria for infection

Screening for suspected infection was carried out by the research team at the place of birth, in the village health worker (VHW) or parent’s home, hospital, or health centre on every participating infant at day 1 and day 90 following delivery, as well as any infant with suspected illness in the interim (day 2–89). A day 1 screening form or interim visit/day 90 screening form which incorporated the WHO Integrated Management of Childhood Illness (IMCI) criteria for possible infection was utilised (Fig. 1) [29]. The use of a validated screening form standardised the information gathered, to identify what constituted an infected child and thereby prevent bias due to researchers subjectively diagnosing infection. The VHWs were trained for 1 day in the protocol and use of the screening form at the start of the study, and training was re-capped each time they returned a completed screening form to the research assistants. The IMCI screening form was utilised as the gold standard for infection data collection, allowing comparison with the other methods of detection.

A combination of passive and active surveillance methods was used to obtain the greatest amount of data possible. Participating women could contact the research team or an allocated VHW following delivery or if they suspected illness in their baby at any time postnatally, whilst the VHW and research team actively contacted the women around the expected due date for delivery, and 90 days from the day of birth. VHWs were encouraged to visit the mothers and complete the screening form twice weekly in the first month, and then weekly thereafter, although this was often not done. Midwives at the local health centres also notified the research team of inpatient births.

Screening data was collected electronically on handheld Samsung Galaxy S4 mobile smart phones using an electronic data capture system—the ‘Open Datacollect Kit’ (ODK). Researchers scanned the participants’ individual barcode or entered their ID manually into the appropriate electronic form, prior to conducting the interview. On completion, the form was encrypted and transferred (via a server in Mbale) in real time to a data analyst at the University of Liverpool.

Any positive response to a question was considered an indicator of infection in the infant, and they were appropriately referred to the local health centre or Mbale Regional Referral Hospital (MRRH) where a clinical diagnosis, samples for culture, and treatment could be obtained. Where possible, clinical notes delineating the physician diagnosis and any associated microbiological evidence were then collected for all infants admitted.

Where expertise and facilities allowed, blood samples were collected, alongside stool and pus samples where relevant. Samples were analysed at the microbiology laboratory of Busitema University. Blood was aseptically collected into BACTEC bottles using the vacutainer system by trained laboratory personnel and loaded with inoculum into the BACTEC 50 blood culture system for auto-detection of microbial growth. Those with positive growth were sub-cultured on MacConkey agar (Oxoid-UK), blood agar, and chocolate agar (Oxoid-UK) for maximum recovery of bacteria. A portion of the stool samples were inoculated in selenite F broth, incubated at 37 °C for 18 h, and then sub-cultured on XLD agar and MacConkey agar. The other portions were directly inoculated on XLD agar and MacConkey agar. Pus swabs were inoculated on blood agar, MacConkey agar, and chocolate agar. All the inoculated plates were incubated at 37 °C for 18–24 h under ambient air except for chocolate agar plates which were incubated at 37 °C in an anaerobic jar. Isolated bacteria were identified using standard biochemical methods and sensitivity tested against a range of locally available antibiotics. Samples were not obtained from infants with antibiotic exposure prior to health centre admission as there is a low detection rate with such samples.

Data was collected on the use of antibiotics. The BabyGel pilot trial interim screening form specifically asked respondents to name any medicines that had been used, and VHWs and/or other health workers were also asked to comment on antibiotic use in free text on the screening tool. Clinical notes were also reviewed to collect written data on drugs administered to the infant during and prior to admission. When mothers retrospectively commented on antibiotic use, they were asked if they had kept the box or prescription for confirmation, or if they could recall the drug administered to the child; cases of uncertainty were not counted, e.g. ‘baby was taken to a nearby drug shop and dispensed unknown syrup’.

The same screening form was used at day 90 and during the interim period; it questioned: ‘Has your baby been unwell since the last visit?’, Has your baby ever been admitted to hospital since the last visit?’, ‘How many times?’ (for each), and ‘For how long did your baby spend in the hospital?’. These questions were designed to capture any infants who had been ill but had screened negative or in whom clinical notes were not collected. Further details to elaborate were also recorded in a free-text box completed by the interviewer at the end of the form.

Finally, an international standard verbal autopsy questionnaire was utilised to determine the cause of death in any infant that died during the study.

### Ethical considerations

This secondary analysis was part of the BabyGel pilot trial, granted ethical approval by the ‘University of Liverpool Research and Ethics Committee’ in the UK, the ‘MRRH Institutional Review Committee’ in Mbale, and the Uganda National Council for Science and Technology (RETH000808, REIRC IN – COM 011/2015 and HS1768 respectively).

Participant consent interviews were conducted in the language most comprehensible to the subject (local language Lumasaba or English), and written consent was subsequently obtained.

## Results

A total of 103 mothers consented to participate in the study (104 infants—including one set of twins). Three stillbirths, an early neonatal death with no screening, and a mother who withdrew from the study with no screening were all excluded; one late neonatal death with completed day 1 screening details and a mother-infant pair who relocated after completing the day 1 screening were retained in the statistical analysis. Thus, a total of 98 women (99 mother-infant pairs) were included, with 96 women (97 mother-infant pairs) completing 3 months of follow-up (Table 1). The mean age of the women who participated in the study was 24.9 years (mean (s.d.) 5.7 [15–38]).

**Table 1:** Demographics of study participants

	*N* (%)
Total participants	103
Age*	
15–20	25 (24.3)
21–30	56 (54.4)
31–40	21 (20.4)
Mean age	24.9
Marital status	
Single	22 (21.4)
Married	80 (77.7)
Divorced/separated	0
Widowed	1 (1.0)
Highest level of education	
No formal education	3 (2.9)
Did not complete primary education	52 (50.5)
Completed primary (PLE)	32 (31.1)
Completed ordinary or advanced level (UCE/UACE)	14 (13.6)
Completed diploma or degree	2 (1.9)
Primary occupation	
Peasant farmer, no paid employment, or housewife	95 (92.2)
Student	3 (2.9)
Business woman	2 (1.9)
Professional	1 (1.0)
Others	2 (1.9)

*Data missing on 1 woman

**Fig. 2:**
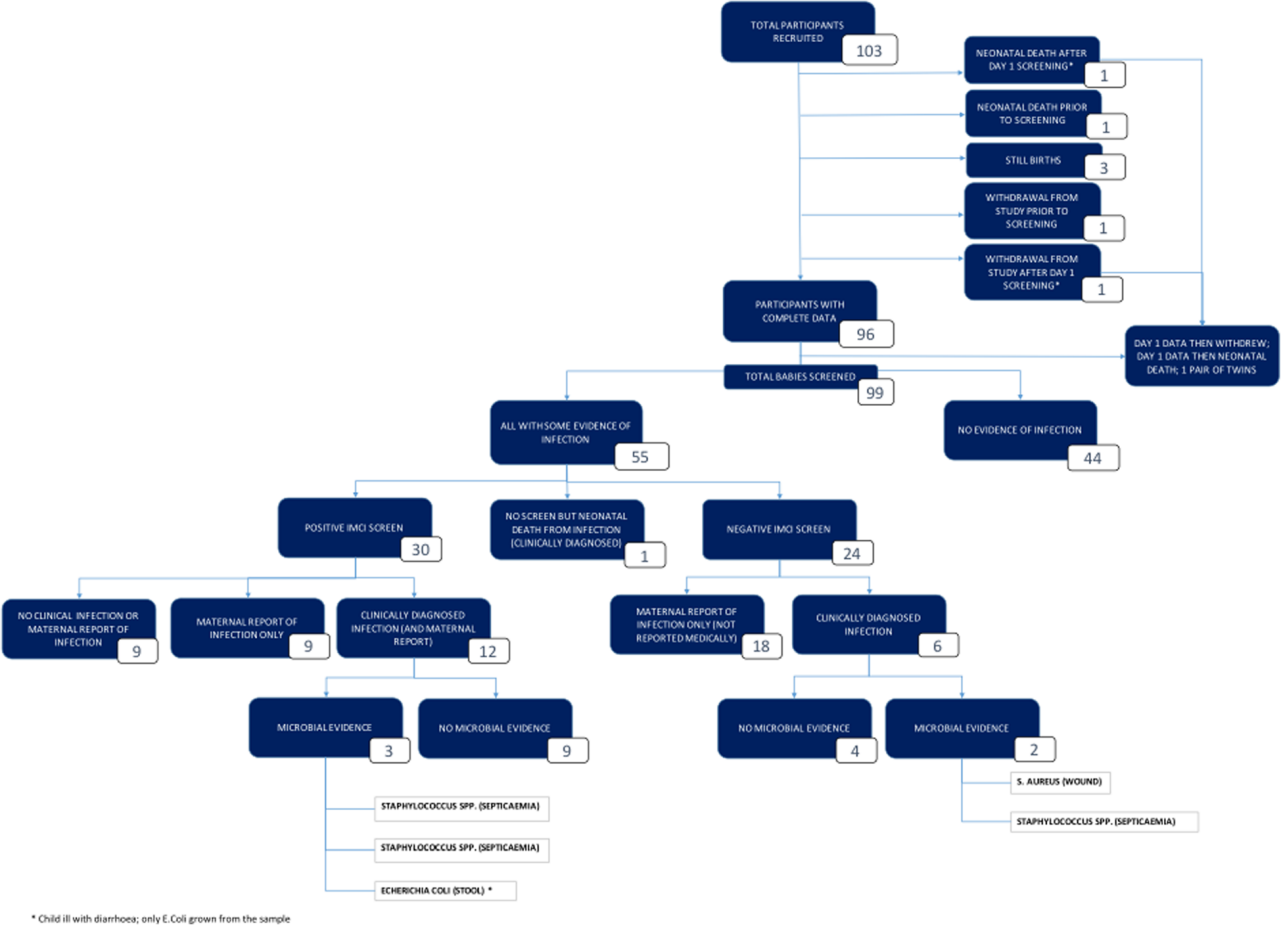
A flow diagram to illustrate the study results

**Fig. 3:**
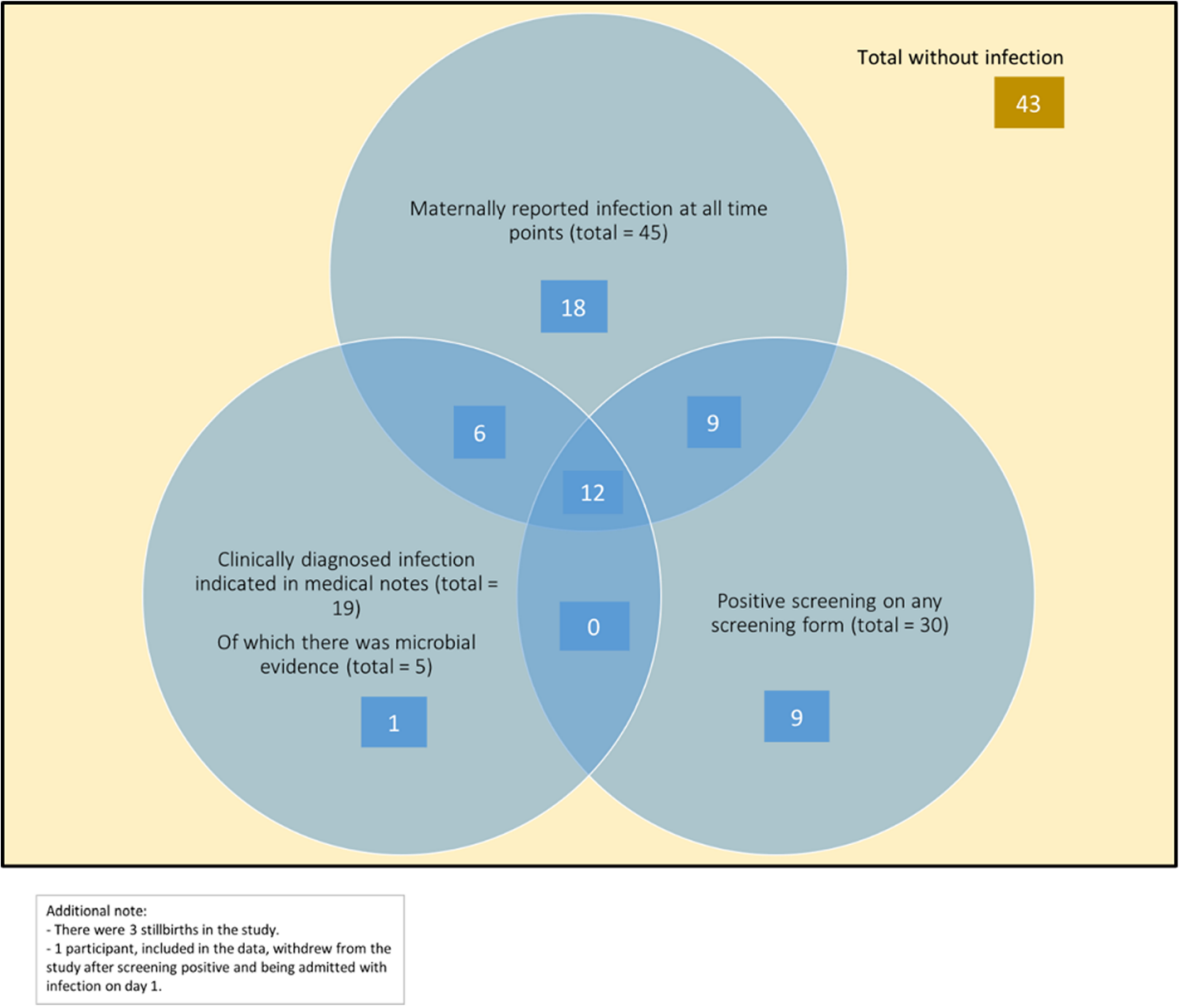
Venn diagram indicating the various sources by which presumed infection was identified in participants

The study identified evidence for suspected infection in 55 (55.6%) of the 99 infants followed up, of which 27 and 28 infants were in the control and interventions arms respectively. The clinical comparative outcomes are reported elsewhere. Figure 2 summarises the results of the study. Considering the 99 infants followed up, in total, 45 infected infants (45.5%) were identified by maternal report, 30 (30.3%) screened positive, and clinical notes were obtained for 19 (19.2%) (of which 5 cases (5.1%) were supported by microbiological evidence). In some cases, infection was positively identified from multiple sources; however, evidence from all three of these sources was only obtained for 12/55 (21.8%) of the total infants identified with suspected infection (Fig. 3). There were no infection outbreaks in the region at the time of the study.

Of the 55 infants with evidence of suspected infection, IMCI screening forms were positive for indicators of infection for 30 infants (54.5%); 22 (40%) infants had a single positive screen and 8 (14.5%) had two positive screens during the follow-up period (mean (s.d.) number of positive screens per infant: 1.27 (0.45)). There were 8 positive screens at the day 1 follow up, 3 at the day 90 follow up, and 27 screened positive for suspected infection during the interim period (Table 2). Reliable dates were obtained for only 11 of the 27 positive screens during the interim period; of these, 2 (18.2%) occurred in the first 14 days after birth, 4 (36.4%) occurred between day 15 and day 30, and the remaining 5 (45.5%) during months 2 and 3 of the follow-up period (mean (s.d.) age of infants at positive screen: 40 (32) days). A relapse was considered any second positive screen < 7 days after the first positive screen, and a new episode of infection was any second positive screen > 7 days after the first. Of the 8 babies with two positive screens during the study, 3 screened positive at day 1 and during the interim (2 unreliable dates and 1 relapse), 2 screened at day 90 and during the interim (1 unreliable date and 1 episode of new infection), and 3 screened positive twice during the interim period (2 unreliable dates and 1 relapse).

**Table 2:** Results of the study for all participants

	*N*	% (95% CI)
Total maternal recruits	103	
Withdrawals	2	
Stillbirths	3	
Neonatal deaths	2	
Total infants screened + followed up*	99	
Any evidence of infection	55	55.6 (45.2:65.5)
Positive screen		
Day 1	8	8.1 (3.6:15.3)
Interim	27	27.3 (18.8:37.1)
Day 90	3	3.0 (0.6:8.6)
Day 1 and/or interim and/or day 90	30	30.3 (21.5:40.4)
Clinical diagnosis of infection (from clinical notes)	19	19.2 (12.0:28.3)
Oomphalitis	1	1.0 (< 0.1:5.5)
Pneumonia	2	2.0 (0.2:7.1)
Diarrhoea	1	1.0 (< 0.1:5.5)
Neonatal septicaemia	6	6.1 (2.3:12.7)
Upper respiratory tract infection	3	3.0 (0.6:8.6)
Skin pustules/infection	3	3.0 (0.6:8.6)
Evidence from all three sources	12	12.1 (6.4:20.2)
Unspecified source	3	3.0 (0.6:8.6)
Microbiological evidence	5	5.1 (1.7:11.4)
Maternally reported infection	45	45.5 (35.4:55.8)
Received antibiotics	22	22.2 (14.5:31.7)
Infants receiving antimalarial medication	8	8.1 (3.6:15.3)

*CI* confidence interval

*Includes 1 set of twins, 1 neonatal death (screened at day 1), and 1 withdrawal (screened at day 1)

Table 3 illustrates the various fields which indicated infection in infants on each screening form. The remaining 25 infected infants were never captured on any screening form, but were identified via clinical notes and maternal report. In this way, successful use of the IMCI screening form criteria was limited; some of these infants screened negative yet were subsequently referred to a health centre and clinically diagnosed with suspected infection.

**Table 3:** Criteria indicating presumed infection in infants (based on IMCI screening forms)

Day	Difficulty feeding	Convulsions	Moving only when stimulated /not at all	Chest wall indrawing	Fast respiratory rate	High axillary temperature	Low axillary temperature
1	5	0	0	0	0	4	0
90	1	1	1	1	0	2	0
Interim (day 2–89)	9	3	2	5	19	11	1
Totals	15	4	3	6	19	17	1

Medical notes were collected for 19 infants who were clinically diagnosed with infection (Table 2), of which 12 had also screened positive for indicators of infection on a screening form (Fig. 3). The VHWs and study researchers could not attend for referral with all infants who screened positive, limiting the amount of medical notes that could be traced. One neonate died within the first month. The suspected cause of death was ‘sepsis’ based on the information gathered in the clinical notes and verbal autopsy form.

It was not possible to obtain lab samples for all 19 infants due to limited resources and staff at the health centres. In total, 16 culture samples were obtained from 10 of these infants, of which 5 samples returned positive results (Table 4). The most common pathogen cultured was *Staphylococcus spp.* (60%), and both *Escherichia coli* and *Staphylococcus aureus* were cultured once each (20% respectively). As anticipated, positive cultures were only obtained in 31% of samples: 3 blood samples, 1 stool sample, and 1 pus sample. No CSF samples were drawn (due to limited resources at the point of care). In cases whereby multiple samples were taken from infants (e.g. blood and pus), growth only occurred in 1 sample.

**Table 4:** Outcome for culture samples obtained

	Organisms	*N* (%)
Total samples taken		16
Blood samples		10 (62.5)
Pus swab		2 (12.5)
Stool sample		4 (25.0)
Cerebrospinal fluid		0
Total positive samples		5 (31.3)
Positive blood cultures	*Staphylococcus spp.* (3)	3 (18.8)
Positive pus swabs	*Staphylococcus aureus* (1)	1 (6.3)
Positive stool samples	*Escherichia coli* (1)	1 (6.3)
Cerebrospinal fluid		0

A total of 45 mothers reported that their child had been infected on the 90-day IMCI screening form or interim form. Of these, 21 infants also screened positive on a screening form; therefore, the question captured 24 cases whereby infants may have been ill but were not additionally screened when ill/did not screen positive on a screening form (Fig. 3). Mothers reported 106 counts of illness in total, due to some infants falling ill on multiple occasions. The IMCI screening forms therefore failed to identify 69 occasions in which infants may have been infected. Data collected via maternal reports of infection identified 45/55 (81.8%) of the total infants with suspected illness.

There is evidence for 22 infants being administered antibiotics, in some cases prior to hospital admission. Table 5 indicates which infants were subject to antibiotic use and where this information was gathered from, and it also lists the types of antibiotics in use and in what frequency used. In total, 8 infants were also clinically diagnosed with malaria (Table 2). All were in the post-neonatal period and received antimalarial medication (14.5% of the total with suspected infection); 3 cases were confirmed with a rapid diagnostic screening test, but there was no evidence of diagnostic testing for the other 5 infants. Co-infection with pneumonia occurred in 1 infant, and a skin infection in another.

**Table 5:** A table indicating antibiotic use in all infants with suspected infection

	*N*	%
Total participants with infection	55	100
Total antibiotic exposure	22	40.0 (27.0:54.1)
Sources of evidence for antibiotic use		
Clinical notes	17	30.9 (19.1:44.8)
Direct question addressing use of any medication on IMCI form	3	5.5 (1.1:15.1)
Comments on antibiotic use (free text)	19	34.5 (22.2:48.6)
Total antibiotic exposures	52*	100
Named antibiotic		
Amoxicillin	8	15.4 (6.9:28.1)
Ceftriaxone	10	19.2 (9.6:32.5)
Gentamycin	7	13.5 (5.6:25.8)
Metronidazole	3	5.8 (1.2:15.9)
Ampiclox	4	7.7 (2.1:18.5)
Nalidixic acid	1	1.9 (0.1:10.3)
Ampicillin	4	7.7 (2.1:18.5)
Cloxacillin	2	3.8 (0.5:13.2)
Benzyl penicillin	2	3.8 (0.5:13.2)
Co-trimoxazole	6	11.5 (4.4:23.4)
Neomycin	1	1.9 (0.1:10.3)
Amikacin	1	1.9 (0.1:10.3)
Mupirocin	1	1.9 (0.1:10.3)
Tetracycline	1	1.9 (0.1:10.3)
Erythromycin	1	1.9 (0.1:10.3)

*Many babies had more than 1 antibiotic

A total of 15 mothers were known to carry HIV, of which 4 were on co-trimoxazole prophylaxis. None of the babies of HIV-infected women received prophylaxis. The infection rate in babies of HIV-infected mothers was 73% compared to 57% in those of non-infected mothers (*p* = 0.236, chi-squared test).

In order to calculate the sensitivity and specificity, clinical diagnosis was utilised as the gold standard (despite there being 80.8% of missing results), assuming that all missing results were negative. With these provisos, the results were:

Maternally reported infection: sensitivity 18/19 (94.7%: 74.0% to 99.9%), specificity 53/80 (66.3%: 54.8% to 76.4%); positive IMCI screen: sensitivity 12/19 (63.2%: 38.6% to 83.7%), specificity 62/80 (77.5%: 66.8% to 86.1%); evidence of antibiotic use: sensitivity 17/19 (89.5%: 66.9% to 98.7%), specificity 75/80 (93.8%: 86.0% to 97.9%).

## Discussion

### Summary of results

Utilising all five methods of data collection, suspected infection was identified in 55.6% of the study participants, a high figure especially when considering some of these infants were ill on multiple occasions. None of the methods of data collection successfully identified all cases of suspected neonatal infection. The IMCI screening forms successfully identified infection in 30.3% of the total study participants and 54.5% of the total number of infants with suspected infection. Evidence for infection from clinical notes was found for only 34.5% of the infants with suspected infection, and microbiological evidence was obtained for only 9.1% of the total suspected with infection. Similar to reports in previous studies, *Staphylococcus* spp. and *Escherichia coli* were the most common causative pathogens identified [2, 12]. Maternal report of infection captured the greatest amount of data, as mothers reported illness for 81.8% of the total presumed infected infants. Finally, evidence for antibiotic use was only found in 40% of the total infected infants.

Whilst it is desirable to have the sensitivity and specificity for each method of data collection, this had to be calculated (and must also be interpreted) with great caution. The analysis was calculated on all 99 infants (including those who did not test positive) and assumes, probably erroneously, that the absence of a positive result for any test method equated to a negative test result. Furthermore, the evidence available in the clinical notes had to be utilised as the gold standard and that itself is suspect.

### Comparison to other studies

The main difference between this study and others was the prospective rather than retrospective element. A recent cross-sectional study in Eastern Uganda involved conducting face to face interviews with 2237 women who had delivered in the past 12 months, to determine the neonatal mortality rate and influential factors [30]. The retrospective nature of the study meant it was therefore subject to recall bias, and the sole reliance of maternal assessment of ‘danger signs at birth’ (e.g. ‘yellow skin’, ‘difficulty breathing’) was arguably subjective and potentially unreliable. A similar community-based prospective cohort study was carried out in Mbale, Eastern Uganda, to determine perinatal mortality and risk factors of perinatal death for 7 days postnatally in 835 women [9]. There were 34 perinatal deaths (18 excluding stillbirths), none of which were officially registered or had death certificates (again illustrating the difficulty of monitoring infection and NMR using the accepted estimated statistics for the country). Again, the women were interviewed within 4 weeks of delivery to recall their antenatal care attendance, socio-demographic characteristics, and infant mortality. Other studies which considered community infection in neonates generally required the women to attend hospital or antenatal clinics for data collection, due to the difficulty of regularly monitoring infants in the community home.

### Limitations of study

The prospective element of this study meant data had to be collected in real time, and the obstacles to data collection faced in this trial highlight why there is a paucity of published research on rural Ugandan mothers and children. Numerous challenges prevented widespread use of the screening forms. The VHWs were not always notified when infants fell ill and may not have been available to visit the mother at home. This likely resulted in cases whereby infection was missed. Mothers also often bypassed the study screening process, attending hospital or health centres directly with their children without notifying the research team, presumably due to concerns about their baby’s health and to avoid delays in seeking care. Others screened positive on the IMCI form, but there was no record of their subsequent attendance at the study health centres as should have happened *per protocol*. Mothers may have taken their infants to local pharmacies or private clinics to prevent the informal charges that are sometimes demanded by health centre staff. Furthermore, mothers were sometimes absent from home during day 1 or 90 screening, and others failed to notify researchers that their child had been born for some time. All these presented enormous difficulties in collecting accurate, reliable data on infant infection.

Although clinical notes were obtained where possible from hospitals and health centres following referral, delays in researcher notification meant that clinical notes were often no longer traceable. Finite members of staff at the health centres and hospitals were notified of the study, and therefore, the safeguarding of participant notes was limited by staff awareness. This was an important learning point from the pilot study and may have been exacerbated by parental refusal to attend hospital following referral which has been documented in previous community-based studies [20, 31].

Unlike the IMCI screening forms, and clinical or microbiological data which made diagnoses in real time, maternal report of infection captured the greatest number of infants with suspected infection, but was subject to recall bias. Likewise, data obtained on antibiotic exposure was occasionally gathered from free-text comments on the screening forms and therefore also subject to recall bias.

Furthermore, the IMCI form is a validated tool for severe illness, whilst the other methods used in this study are more specific for infections. This makes comparison of infection rates challenging. Mothers were asked: ‘Has your baby been unwell since the last visit?’ ‘How many times has baby been unwell?’ and ‘Has your baby ever been admitted to the hospital since the last visit?’. Besides recall bias, the closed questions neither establish the severity of the suspected infection nor the time period. Thus, it would be possible for the quoted rates to include both minor infections and relapses rather than individual episodes of severe illness. Only 18 of the 45 infants (40%) identified via maternal report were clinically diagnosed. This can be partially explained by lack of care-seeking at health facilities and the difficulty in collecting clinical documentation from busy health centres and might also indicate limitations in maternal recognition and report of severe infection.

Collecting hard evidence for infant infection was challenging. Whilst clinical notes reported infections ranging from neonatal septicaemia to skin pustules and diarrhoea, microbiological evidence for infection was very limited. Although funding for tests was made available for the study, established local practice is not to rely on microbiological testing but rather to provide empirical treatment. Furthermore, in Mbale, antibiotics can be obtained with ease over the counter from local sources without a qualified diagnosis being made. Antibiotic use could therefore not be confidently utilised as an indicator of infection (and especially severe infection) in participants, even though the information was easily obtained retrospectively from mothers.

The free-text comments documented on the screening forms indicated liberal use of freely available antibiotics within the rural setting without a prescription. Antibiotic exposure prior to admission meant limited samples could be obtained from infants, resulting in few cases of illness with microbiological evidence. Although 31% of culture samples obtained were positive (comparable to the 35 and 37% obtained in the Mbarara Regional Referral Hospital and Mulago Hospital studies respectively) [2, 12], only 10 infants with suspected illness had samples taken for culture. Furthermore, the process of transporting samples was not without error: one baby had samples taken for complete blood count (CBC) with blood culture and sensitivity which was delivered to the hospital where there were no personnel to receive it. The sample subsequently haemolysed and was rejected due to a public holiday resulting in the sample remaining at room temperature for > 48 h.

A further reason for the limited number of samples was the lack of skilled staff. Hospitals and health centres in Uganda are often under-staffed, and therefore, necessary tests and investigations cannot be carried out as required, or advised by local protocol. Arguably, this is one of the greatest challenges of carrying out research of this type in this environment. With appropriate funding, it would be possible to set up a study in a high-quality European standard paediatric ward with all the associated resources, but this would ultimately alter the study setting and prevent this being a pragmatic study.

### Strengths of study

The study had a high follow-up rate, with all participants being tracked to day 90 postnatally, except the women who withdrew their consent or relocated and those with perinatal deaths. The prospective format gives a variety of strengths: the potential for clear temporal chronology to link the outcome to the exposure, limited risk of recall bias due to rapid assessment with the IMCI screening forms, and clinical or microbiological diagnosis in real time. The variety of methods utilised for data collection meant that a spectrum of illness was captured. In cases whereby multiple methods successfully identified singular episodes of infection, the combined data was complementary, creating a sound clinical picture, which could be utilised to differentiate severe from minor infection.

### Future recommendations

In future studies, alterations must be made to the methods of data collection and the details gathered. The choice of method will depend on a balance of cost, feasibility, and acceptability. Unless all methods measure severe infection only, differences in infection risk will be incomparable; limiting the study outcome to major infections would permit comparative analysis. The IMCI screening form should only identify severe infection, as should the clinical notes of those presenting directly to health care providers. In contrast, the maternal report of infection captured a huge number of infants with potentially minor or suspected infections. This was compounded by the difficulty of VHWs accessing the community homes making it difficult to verify maternal reports. In order to reduce recall bias and ensure the infections being identified are severe, the maternal questionnaire about recent infections should include greater detail on the symptoms, signs, date, and length of illness. Regular, weekly communication with participants via a toll-free number would improve communication between the researchers and mothers to determine if the child has been ill in the past week (whilst also reducing the risk of recall bias). A researcher could attend the home to assess the ill child or visit the health centre/hospital the mother attended during the week to collect clinical notes if the baby was admitted, to ensure as many infants with suspected infection are captured as possible. Particular attention should be paid around the expected date of delivery and final follow-up, with frequent communication to guarantee adequate data collection.

To improve the collection of both clinical and microbiological data, improved communication between the health centres and laboratory alongside more reliable services will be required for future studies. Although multiple samples were obtained from infected infants, only singular samples yielded results, confirming the importance of obtaining multiple samples where possible and relevant. This thorough method optimised the chance of achieving measurable pathogenic growth and should continue to be utilised in future studies. As many members of clinical staff as possible should be notified of the research taking place at the health centres and local hospital, so they have an awareness of the mothers participating and can notify researchers, keeping notes and samples relating to the participants safe. Where screening forms result in a referral to a health centre or hospital, researchers should attend with the infant in order to collect the clinically relevant notes and make the health practitioners aware that the infant is participating in the study.

The challenge of antibiotic use prior to hospital admission and self-prescribed antibiotic use within the community is difficult to address. During the 90-day interview, numerous mothers described occasions on which their child had been ill, and they had self-medicated them and/or not notified the VHWs. Future studies could monitor antibiotic use reported both verbally and with evidence of prescription or drug packaging to more accurately capture the cohort of infants with suspected infection. Administration of antibiotics is a useful indicator of how ill the mother deems their child to be, particularly as a limitation of maternal report of infection is the risk of identifying minor illness rather than severe infections. The benefit of capturing data through maternally reported antibiotic use includes identifying suspected infection regardless of where the care was sought and would be helpful to monitor as a public health outcome given the financial cost of antibiotic use and development of antibiotic resistance, which is a growing international concern [20, 32]. Drawbacks include a potential overestimation of the frequency of true infections and difficulty in ascertaining which drugs were administered as often drugs are provided over the counter without a prescription. This might be addressed by ensuring prescriptions and packaging are retained for inspection by the research team. Alternatively, to reduce the risk of overestimation, evidence for antibiotic use could be restricted to that provided by qualified health care professionals. The downside would be that it might also result in cases being missed. There is currently a paucity of data on the use of antibiotics within Uganda’s rural communities [33], and monitoring this in future would be valuable [34, 35].

## Conclusions

Rural communities are often relatively inaccessible and isolated, and antibiotics are frequently obtained over the counter without a prescription. Formal clinical diagnoses and laboratory-based evidence are rare, due to antibiotic use prior to admission and limited medical and laboratory facilities. There were limitations to each method of infection ascertainment, yet overall, each method captured some evidence for suspected infection, in some cases creating a clinically complementary picture. It follows that each method of data collection utilised in this study could be beneficial if used in future studies with some considerations. Future studies should focus on the clinical diagnosis of severe infection. The IMCI screening tool is a validated method of picking up severe illness and should continue to be used as a means of detecting community infection and directing referral, with clinical diagnosis being used for confirmation. Screening should be improved with weekly contact with the mothers to detect maternally suspected illness. Greater detail of the symptoms and timescales would enhance the accuracy of detection and enable maternal reports of infection to more confidently indicate the total burden of suspected disease of relevant severity. Finally, communication between researchers, health care staff, and participants is crucial, so as to provide high-quality data on infective morbidity.

## Abbreviations

CBC: Complete blood count
CSF: Cerebrospinal fluid
IMCI: Integrated Management of Childhood Illnesses
MRRH: Mbale Regional Referral Hospital
NMR: Neonatal mortality rate
ODK: Open Datacollect Kit
PCR: Polymerase chain reaction
VHW: Village health worker
WHO: World Health Organization
YICSSG: Young Infant Clinical Signs Study Group
